# Omega-3 meat enrichment and *L-FABP*, *PPARA*, and *LPL* genes expression are modified by the level and period of tuna oil supplementation in slow-growing chickens

**DOI:** 10.1093/jas/skad267

**Published:** 2023-08-07

**Authors:** Wichuta Khosinklang, Satoshi Kubota, Cindy Riou, Pramin Kaewsatuan, Amonrat Molee, Wittawat Molee

**Affiliations:** School of Animal Technology and Innovation, Institute of Agricultural Technology, Suranaree University of Technology, Nakhon Ratchasima 30000, Thailand; School of Animal Technology and Innovation, Institute of Agricultural Technology, Suranaree University of Technology, Nakhon Ratchasima 30000, Thailand; School of Animal Technology and Innovation, Institute of Agricultural Technology, Suranaree University of Technology, Nakhon Ratchasima 30000, Thailand; School of Animal Technology and Innovation, Institute of Agricultural Technology, Suranaree University of Technology, Nakhon Ratchasima 30000, Thailand; School of Animal Technology and Innovation, Institute of Agricultural Technology, Suranaree University of Technology, Nakhon Ratchasima 30000, Thailand; School of Animal Technology and Innovation, Institute of Agricultural Technology, Suranaree University of Technology, Nakhon Ratchasima 30000, Thailand

**Keywords:** feeding period, Korat chicken, meat quality, omega-3 polyunsaturated fatty acid, slow-growing chicken, tuna oil

## Abstract

This study proposes a strategy to manipulate the fatty acid (**FA**) content in slow-growing Korat chicken (**KRC**) meat using tuna oil (**TO**). To determine the optimal level and feeding period of TO supplementation, we conducted a study investigating the effects of dietary TO levels and feeding periods on meat quality, omega-3 polyunsaturated fatty acid (**n-3 PUFA**) composition, and gene expression related to FA metabolism in KRC breast meat. At 3 wk of age, 700 mixed-sex KRC were assigned to seven augmented factorial treatments with a completely randomized design, each consisting of four replicate pens containing 25 chickens per pen. The control group received a corn-soybean-based diet with 4.5% rice bran oil (**RBO**), while varying amounts of TO (1.5%, 3.0%, or 4.5%) replaced a portion of the RBO content in the experimental diets. The chickens were fed these diets for 3 and 6 wk, respectively, before being slaughtered at 9 wk. Our results indicated no significant interactions between TO levels and feeding periods on the growth performance or meat quality of KRC (*P* > 0.05). However, the liver fatty acid-binding protein gene (***L-FABP***, also known as *FABP1*), responsible for FA transport and accumulation, showed significantly higher expression in the chickens supplemented with 4.5% TO (*P* < 0.05). The chickens supplemented with 4.5% TO for a longer period (3 to 9 wk of age) exhibited the lowest levels of n-6 PUFA and n-6 to n-3 ratio, along with the highest levels of eicosapentaenoic acid, docosahexaenoic acid, and n-3 PUFA in the breast meat (*P* < 0.05). However, even a short period of supplementation with 4.5% TO (6 to 9 wk of age) was adequate to enrich slow-growing chicken meat with high levels of n-3 PUFA, as recommended previously.

Our findings indicated that even a short period of tuna oil supplementation could lead to desirable levels of omega-3 enrichment in slow-growing chicken meat. This finding has practical implications for the poultry industry, providing insights into optimal supplementation strategies for achieving desired FA profiles without adversely affecting growth performance or meat quality.

## Teaser

Tuna oil supplementation can enhance the fatty acid profile of breast meat in a slow-growing chicken. Additionally, high levels of tuna oil supplementation lead to increased expression of the liver fatty acid-binding protein gene, which could potentially serve as a genetic marker for monitoring the accumulation of beneficial omega-3 polyunsaturated fatty acids and evaluating the meat quality in slow-growing chickens.

## Introduction

Slow-growing chicken play a vital role as a valuable source of high-quality proteins and contribute to household income (Maliwan et al., 2017). These chickens are primarily raised by small or medium-scale farmers, particularly in developing countries, who often face challenges competing with larger enterprises due to their limited competitiveness (Wilson et al., 2022). Despite their relative low productivity, the meat of slow-growing chickens exhibits desirable attributes such as a higher proportion of lean muscle, lower fat content, and improved overall meat quality compared to commercial breeds, leading to a higher demand for slow-growing chicken (Katekhaye, 2019).

In recent years, there has been an increasing consumer demand for natural and functional meats containing specific compounds that promote health and well-being (Supachaturat et al., 2017). Omega-3 polyunsaturated fatty acids (**n-3 PUFA**) have been recognized for their potential to prevent cardiovascular diseases and other degenerative conditions (Palmquist, 2009). However, since the human body cannot produce n-3 PUFA, it is necessary to obtain these beneficial compounds directly from food sources. Hence, to meet this growing demand for food consumption, the present study focuses on enriching chicken meat with n-3 PUFA, aiming to offer high-value and distinctive products to the market. Our strategy for the development of slow-growing chicken aligns with national strategies aimed at enhancing farmers’ competitiveness and supporting sustainable development goals (**SDGs**) such as SDG1 (no poverty), SDG2 (zero hunger), and SDG3 (good health and well-being) established by the United Nations (UN, 2018).

Numerous studies have demonstrated the potential to enhance the n-3 PUFA content in chicken meat through dietary supplementation with various sources such as tuna oil (**TO**), fish oil (**FO**), and certain plants oils (Poureslami et al., 2010; Yang et al., 2010). In line with these findings, our previous study achieved similar results, indicating that a diet supplemented with 4.0% TO to chicken (3 to 12 wk of age) produced “high n-3 PUFA meat” (Hang et al., 2018). Furthermore, a study revealed that the expression of different genes involved in n-3 PUFA biosynthesis influences the accumulation of these fatty acids (**FA**) in chicken meat (Haug et al., 2014). Boschetti et al. (2016) reported higher expression of fatty acid desaturase (**FADS**) genes *FADS1* and *FADS2*, involved in n-3 and omega-6 polyunsaturated fatty acids (**n-6 PUFA**) metabolism, in slow-growing chickens, resulting in increased n-3 PUFA content in breast meat compared to fast-growing chicken breeds.

Although it is established that dietary supplementation can successfully enrich chicken meat with n-3 PUFA, the influence of the supplementation period on n-3 PUFA accumulation remains unclear. Specifically, the impact of the TO supplement period on n-3 PUFA accumulation in slow-growing chicken meat has not been investigated yet. Previous studies have indicated that supplementation with FO, flaxseed, and rapeseed for more than 2 wk before slaughtering could enhance n-3 PUFA content in meat (Konieczka et al., 2017). Furthermore, Zuidhof et al. (2009) demonstrated that supplementing dietary ground full-fat flaxseed for 24.1 d is the most cost-effective approach to achieve n-3 PUFA enriched-chicken meat. It is recognized that the duration of feed retention impacts hormones involved in triglyceride and FA metabolism, thus influencing FA modification and accumulation (Latshaw, 2008). Based on this understanding, we hypothesized that both the level and period of dietary supplementation may significantly contribute to the accumulation of n-3 PUFA in slow-growing chicken meat.

When chickens consume a diet rich in n-3 PUFA, the FA are broken down in the small intestine by pancreatic lipase and transported as very-low-density lipoprotein to be stored in muscle and adipose tissue (Hulver et al., 2003). It was previously reported that various genetic and environmental factors influence fat accumulation in chicken meat (Nie et al., 2015). De Tonnac et al. (2016) showed that different dietary FA can affect gene expression related to lipogenesis and de novo synthesis of long-chain FA. Among the genes involved in fat metabolism, liver fatty acid-binding proteins (***L-FABP***) are essential in lipid transport (Yuan et al., 2012). n-3 PUFA can enhance peroxisome proliferator-activated receptor alpha (***PPARA***) gene, which controls peroxisomal β-oxidation and the conversion of tetracosahexaenoic acid (**C24:6n-3**) to docosahexaenoic acid (**DHA**; **C22:6n-3**) (Royan et al., 2011). Lipoprotein lipase (***LPL***) plays a crucial role by providing substrates for lipid storage in broiler meat (Enerback and Gimble, 1993). Previous studies have identified that the *L-FABP*, *PPARA*, and *LPL* genes are three key regulators of lipid metabolism. Therefore, an investigation focusing on molecular mechanisms underlying fatty acid metabolism and accumulation in slow-growing chicken meat is necessary.

The present study aims to investigate the effect of varying levels and feeding periods of dietary TO supplements on meat quality, n-3 PUFA, and the expression of critical genes involved in FA metabolism. For this purpose, slow-growing Korat chickens (**KRC**), a crossbreed between Thai indigenous Leung Hang Khao males (**LHK**) and Suranaree University of Technology (**SUT**) line females, was utilized for the experimentation. The findings from this research can provide new and valuable insights into determining the optimal level and period of TO supplementation, facilitating the development of targeted feeding strategies to enhance the functional value of chicken meat.

## Materials and Methods

### Ethics statement

The experimental protocols conducted in this study were approved by the Ethics Committee on Animal Use of SUT, located in Nakhon Ratchasima, Thailand (user application ID: U1-02633-2559).

### Birds, experimental design, and diets

A total of 700 1-d-old, mixed-sex indigenous Thai crossbred KRC (1:1 male-to-female ratio) were obtained from the poultry research unit of SUT Farm and raised according to the farm’s guidelines. The chickens were housed in floor pens with a stocking density of eight birds per square meter in an open-housing system.

Before the start of the experiment, the chickens were fed with 21% of crude protein (**CP**) diet that contained 3,100 kcal/kg of ME. Chickens at 3 wk of age with an average weight of 245.82 ± 2.04 g were assigned to an augmented factorial experiment with a completely randomized design. The experiment comprised seven treatments, with four pens assigned to each treatment and 25 birds housed in each pen.

The control group received a corn-soybean-based diet supplemented with 4.5% rice bran oil (**RBO**) from Thai Ruam Jai Korat Co. Ltd, Nakhon Ratchasima, Thailand. In the experimental groups, a portion of the RBO content was replaced with TO at different ratios: 1.5% TO + 3.0% RBO, 3.0% TO + 1.5% RBO, or 4.5% TO + no RBO. All experimental diets contained dl-α-tocopheryl acetate at 200 mg/kg. The chickens were fed these diets for either 3 or 6 wk before being slaughtered at 9 wk. Throughout the experimental period, the chickens had unlimited access to water and feed. All experimental diets were formulated to have equal protein levels (19% CP in the grower diet and 17% CP in the finisher diet) and energy levels (3,100 kcal of ME/kg), as shown in Table 1.

**Table 1.** Compositions and calculated nutrient contents of starter, grower and finisher diets (g/100 g diet, as-fed basis)

Item	Grower (3 to 6 wk)				Finisher (6 to 9 wk)			
	Control	1.5% TO^1^	3.0% TO^1^	4.5% TO^1^	Control	1.5% TO^1^	3.0% TO^1^	4.5% TO^1^
Soybean meal (44% CP^2^)	33.00	33.00	33.00	33.00	26.84	26.84	26.84	26.84
Corn (7.8% CP^2^)	58.80	58.80	58.80	58.80	65.00	65.00	65.00	65.00
Rice bran oil	4.50	3.00	1.50	-	4.50	3.00	1.50	—
Tuna oil	—	1.50	3.00	4.50	—	1.50	3.00	4.50
dl-Met	0.21	0.21	0.21	0.21	0.14	0.14	0.14	0.14
l-Lys	0.18	0.18	0.18	0.18	0.19	0.19	0.19	0.19
l-Thr	0.02	0.02	0.02	0.02	—	—	—	—
Salt	0.35	0.35	0.35	0.35	0.35	0.35	0.35	0.35
Calcium carbonate	1.42	1.42	1.42	1.42	1.20	1.20	1.20	1.20
Monocalcium phosphate (21% P)	1.02	1.02	1.02	1.02	1.28	1.28	1.28	1.28
Vitamin E	0.0002	0.0002	0.0002	0.0002	0.0002	0.0002	0.0002	0.0002
Premix^3^	0.50	0.50	0.50	0.50	0.50	0.50	0.50	0.50
Calculated nutrients (%)								
ME^4^, Kcal/kg	3,100	3,100	3,100	3,100	3,100	3,100	3,100	3,100
CP	19.00	19.00	19.00	19.00	17.00	17.00	17.00	17.00
Digestible Lys	0.96	0.96	0.96	0.96	0.85	0.85	0.85	0.85
Digestible Met	0.44	0.44	0.44	0.44	0.35	0.35	0.35	0.35
Digestible Met + Cys	0.66	0.66	0.66	0.66	0.55	0.55	0.55	0.55
Digestible Thr	0.74	0.74	0.74	0.74	0.68	0.68	0.68	0.68
Calcium	0.90	0.90	0.90	0.90	0.86	0.86	0.86	0.86
Available phosphorus	0.35	0.35	0.35	0.35	0.39	0.39	0.39	0.39
Analyzed nutrient level, %								
Total lipid, %	7.05	7.20	7.12	7.17	7.10	7.05	7.08	7.19
Fatty acid profile (% of total fatty acid)								
SFA^5^	24.85	25.29	28.11	29.72	23.08	25.26	26.77	32.70
MUFA^6^	36.99	34.42	32.55	33.48	39.69	36.04	35.14	33.20
PUFA^7^	38.16	40.29	39.36	36.8	37.23	38.70	38.09	34.10
SFA^5^/MUFA^6^	0.67	0.73	0.71	0.88	0.58	0.70	0.76	0.98
n-6^8^	36.48	33.59	30.07	25.73	35.67	32.75	28.27	22.47
n-3^9^	1.68	6.70	9.29	11.07	1.56	5.95	9.82	11.63
n-6/n-3 ratio^10^	21.71	5.01	3.24	2.32	22.71	5.49	2.88	1.93

^1^Tuna oil.

^2^Crude protein.

^3^Premix (0.5%) included 11.04 mg of pantothenic acid; 35 mg of nicotinic acid; 1 mg of folic acid; 15 µg of biotin; 250 mg of choline chloride; 60 mg of Mn; 45 mg of Zn; 80 mg of Fe; 1.6 mg of Cu; 0.4 mg of I; 0.15 mg of Se; 15,000 IU of vitamin A; 3,000 IU of vitamin D3; 25 IU of vitamin E; 5 mg of vitamin K3; 2 mg of vitamin B1; 7 mg of vitamin B2; 4 mg of vitamin B6; 25 µg of vitamin B12. Per kilogram of diet.

^4^Metabolizable energy.

^5^Saturated fatty acids.

^6^Monounsaturated fatty acids.

^7^Polyunsaturated fatty acids.

^8^Omega-6 polyunsaturated fatty acids.

^9^Omega-3 polyunsaturated fatty acids.

^10^Omega-6 to omega-3 polyunsaturated fatty acid ratio.

### Growth performance and carcass composition

The parameters used to evaluate chicken growth performance including weekly recordings of body weight (**BW**) and feed intake (**FI**) were weighed and recorded on a pen basis, followed by BW gain (**BWG**) and feed conversion ratio (**FCR**) calculations.

### Sample collection

At 9 wk of age, following a 12-h feed withdrawal period, 84 Korat chickens were transported to the university slaughterhouse. Among them, 56 chickens (2 chickens per pen: one male and one female) were subjected to electrically stunned, scalded, machine de-feathering, and manual evisceration. Breast samples were collected from these chickens and stored at –20 °C for subsequent meat quality and FA profile analysis. Additionally, the remaining 28 chickens (one male per pen) were slaughtered using chloroform for gene expression analysis. Liver and breast muscle tissues were removed, ­immediately immersed in liquid nitrogen, and stored at –80 °C for the subsequent gene expression analysis.

### pH measurement

The pH of the breast meat samples was measured using a pH meter connected to a probe inserted into the center of the pectoralis major muscle. The measurements were taken at 45 min and 24 h after slaughter. Each sample was measured three times at three different locations, and the average value was for statistical analysis.

### Drip loss measurement

Drip loss was measured using pieces of breast meat cut to 1.5 × 3.0 × 0.5 cm^3^ from the exact location. The meat samples were then hung in a storage room at 4 °C. After 24 h, the samples were weighed, and the drip loss was calculated as a percentage of the initial weight using the following formula:


$$\begin{array}{l} Drip\;Loss\left(\%\right)=\frac{\left(Weight_{before\;storage}-Weight_{after\;storage}\right)}{Weight_{before\;storage}}\times 100 \end{array}$$

### Cooking loss measurement

Breast samples were weighed and sealed in plastic bags before being boiled in a water bath until their internal temperatures reached 80 °C. The samples were reweighed, and the percentage of the initial weight was calculated using the following formula:


$$\begin{array}{l} Cooking\;Loss\left(\%\right)=\frac{\left(Weight_{before\;boiling}-Weight_{after\;boiling}\right)}{Weight_{before\;boiling}}\times 100 \end{array}$$

### Shear force measurement

Shear force was measured using a Texture analyzer (TA-XT2, Texture Technologies Corp., Scarsdale, New York, USA.) coupled with a Warner–Bratzler on breast tissue samples that had been preheated in a water bath at 80 °C for 10 min. The samples were then cut into cubes measuring 2.0 × 1.0 × 0.5 cm^3^ and sliced parallel to the muscle fibers. The crosshead was programmed to travel at 20 cm/min.

### Meat thiobarbituric-acid-reactive substance measurements.

The concentration of thiobarbituric-acid-reactive substance (**TBARS**) was determined by measuring the malondialdehyde (**MDA**) concentration, a widely used indicator of ­oxidation. Two pieces of 5 g breast meat samples were analyzed following the modified protocol by Leick et al. (2010). The absorbances of the standards and samples were determined at 532 nm using a Thermo Scientific Multiskan GO Microplate Spectrophotometer (Thermo Fisher Scientific Oy., Vantaa, Finland).

### Fatty acid profile analysis

For lipid extraction, 90 mL chloroform: methanol (2:1, v/v) was used with 5 g of breast sample following the method of Folch et al. (1957). The extracted lipid was then methylated using the technique described by Metcalfe et al. (1966), and the resulting FA methyl esters were analyzed using gas chromatography (7890A; Agilent Technologies, Santa Clara, CA, USA). The analysis employed a flame ionization detector and a capillary column (SP-2560, 100 m × 0.25 mm ID, Supelco Inc., Bellefonte, PA, USA), with helium as the carrier gas at a flow rate of 0.95 mL/min.

The injector and detector were set to a temperature of 260 °C. The column temperature was initially set at 70 °C and increased to 175 °C at a rate of 13 °C/min and finally increased to 240 °C at a rate of 4 °C/min. The FA contents in the breast meat were calculated using the formula reported by Hang et al. (2018).

### Real-time PCR analysis

Total RNA was extracted from the liver and breast muscle using Trizol Reagent (Thermo Fisher Scientific), and the quality of the RNA was determined using a NanoDrop 2000 spectrophotometer (Thermo Fisher Scientific). RNA was converted to cDNA according to the manufacturer’s protocol using a high-capacity cDNA reverse transcription kit (Thermo Fisher Scientific). The Light Cycler 480 system was used to perform qPCR analysis (Roche Diagnostics GmbH, Mannheim, Germany). *L-FABP* and *PPARA* expression levels were analyzed in the liver tissue, while *LPL* gene expression was evaluated in the breast tissue. Glyceraldehyde-3-phosphate dehydrogenase (***GAPDH***) was used to normalize the gene expression data. A qPCR was performed in 20 µL reaction volumes containing 2 µL of cDNA template, 1 µL of each of the forward and reverse primers, 10 µL of LightCycler 480 SYBR Green I Master (Roche Diagnostics GmbH), and 6 µL nuclease-free water. Each gene was analyzed three times. A melting curve analysis was performed to validate the specificity of the PCR fragments. The 2^−∆∆CT^ method was used to calculate the relative gene expression level of each gene to *GAPDH*, as explained by Livak and Schmittgen (2001). The primer sequences for the gene expression analysis are shown in Table 2.

**Table 2.** Primer sequence list for real-time PCR

Gene	Sequence (5ʹ—3ʹ)	Product size	Gene bank accession number
*L-FABP* ^1^	F: GAGCTCCAGTCCCATGAAAA R: TCAGCAGCTCCATCTCACAC	202 bp	AF380999
*PPARA* ^2^	F: CAAACCAACCATCCTGACGAT R: GGAGGTCAGCCATTTTTTGGA	64 bp	NM 001001464.1
*LPL* ^3^	F: TTGGTGACCTGCTTATGCTA R: ATTGCTGCCTCTTCTCCTTT	187 bp	X14670
*GAPDH* ^4^	F: GGTGGCCATCAATGATCCCT R: CCGTTCTCAGCCTTGACAGT	105 bp	NM204305.1

^1^Liver fatty acid-binding protein.

^2^Peroxisome proliferator-activated receptor alpha.

^4^Lipoprotein lipase

^4^Glyceraldehyde-3-phosphate dehydrogenase.

F, forward; R, reverse.

### Statistical analyses

Growth performance, meat quality, and FA data from the breast meat were analyzed using analysis of variance (**ANOVA**) as an augmented 2 × 3 factorial + 1 design. The generalized linear model procedure of SAS (SAS Institute Inc. 2014. SAS OnDemand for Academics. Cary, NC: SAS Institute Inc.) was employed for this analysis. The statistical model considered the control, level, and period of TO supplementation and their interaction. Orthogonal contrasts were applied to compare the control group with the other treatments and to assess the main effect of the period of TO supplementation, as well as the interactions between the levels and periods of TO supplementation. Orthogonal polynomial contrasts were used to examine the linear and quadratic effects of increasing TO supplementation (1.5%, 3.0%, and 4.5%) in the diet. The treatment means were separated using Tukey’s multiple comparison tests and were considered significant at *P* < 0.05. All data are expressed as means ± SEM.

**Figure 1.**
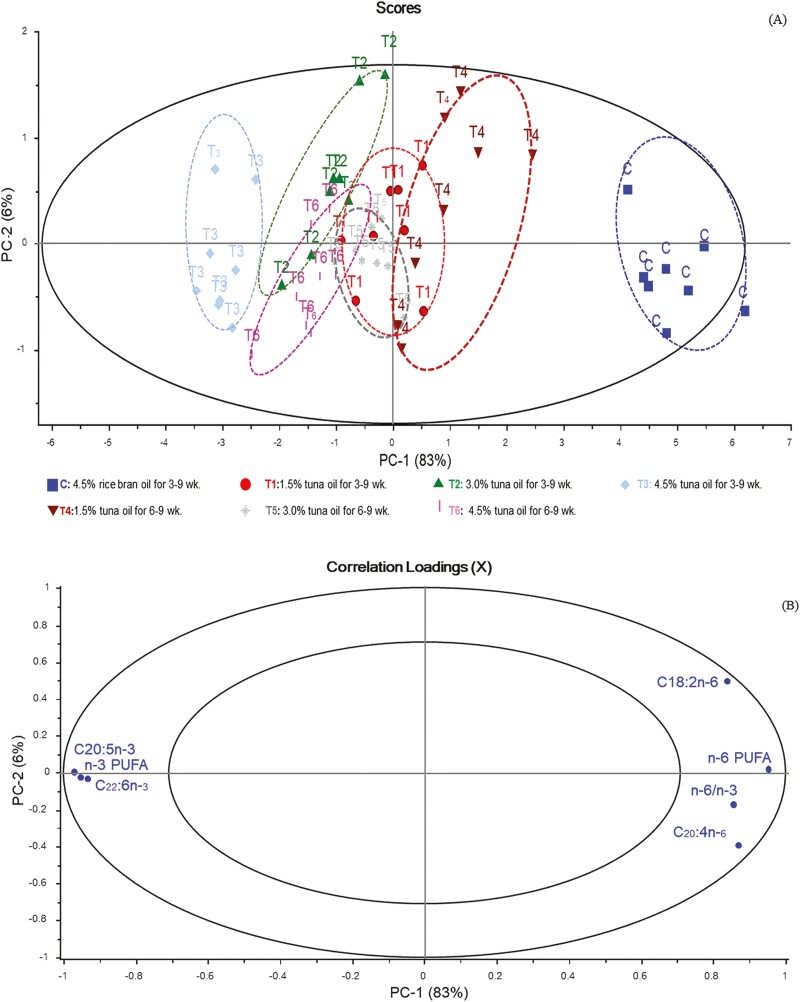
Principal component analysis (PCA) (A) for PC1 vs. PC2 for the seven different experimental data and correlation loading plot (B) for PC1 vs. PC2 for key n-6 PUFA and n-3 PUFA at 83% total variance in Korat chicken breast meat of the different experimental groups with the outer and inner ellipse representing 100% and 50% of the variance, respectively. Abbreviation: C20:5n-3, eicosapentaenoic acid (EPA); C22:6n-3, docosahexaenoic acid (DHA); C18:2n-6, linoleic acid (LA); C20:4n-6, arachidonic acid (AA); n-3, omega-3 polyunsaturated fatty acids; n-6, omega-6 polyunsaturated fatty acids; n-6/n-3, n-6 to n-3 PUFA ratio.

**Figure 2.**
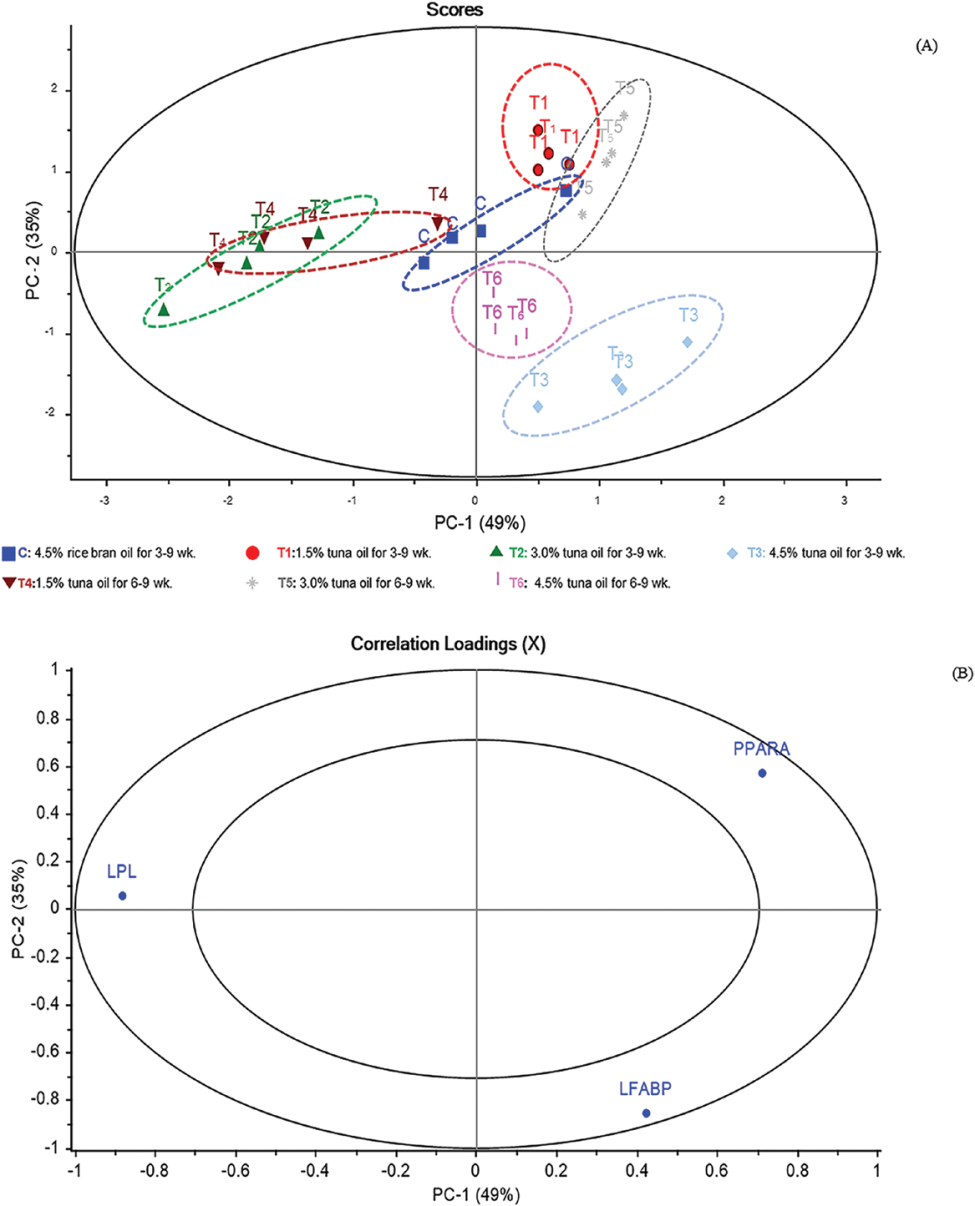
Principal component analysis (PCA) (A) for PC1 vs. PC2 for the seven different experimental data and correlation loading plot (B) for PC1 vs. PC2 for expression of genes involved in fatty acid metabolism at 49% total variance in male Korat chicken breast meat of the different experimental groups with the outer and inner ellipse representing 100% and 50% of the variance, respectively. Abbreviation: *LPL*, lipoprotein lipase gene; *PPARA*, peroxisome proliferator-activated receptor alpha; *LFAB*, liver fatty acid-binding proteins.

In addition, the combination treatments, key FA, and gene expression were measured. The clustering of the variables was analyzed using principal component analysis (**PCA**). A biplot was used to identify the relationships between variables and sample properties. The correlation between n-3 PUFA ­content (Figure 1A and 1B) and gene expression (Figure 2A and 2B) for each cluster of the control and the treatments of the chicken samples in the data matrix were weighted using SD weighting process and calculated using PCA, after which a biplot correlation between variables was created using multivariate analysis.

Furthermore, Spearman’s rank correlation and correlation matrix plots were generated using the corrplot package (Wei and Simko, 2017) in R version 2.1 (R Core Team, 2020) to investigate the relationship between gene expression and key FA content in KRC breast meat.

## Result and Discussion

### Growth performance, meat quality, and meat TBAR

The supplementation of different levels of TO in the diet for varying feeding periods did not show any significant effects on growth performance, meat quality parameters, and meat TBAR compared to the control treatment (*P* > 0.05; Table 3). These findings align with our initial hypothesis, potentially attributed to the addition of TO up to 4.5% in the diet, which did not cause any noticeable odor or affect palatability, thus not negatively impacting the feed intake of KRC. This is consistent with previous studies such as Morales-Barrera et al. (2013), who demonstrated that the inclusion of TO in chicken diet had no adverse effect on chicken performance, and Sadeghi et al. (2012), who found that supplementing chicken diet with up to 5% FO had no impact on FI, BW, or FCR. Additionally, the dietary treatments used in this study were formulated to be isoenergetic and isonitrogenous and supplemented with 200 mg/kg vitamin E, which acts as an antioxidant to against FA oxidation, reduces free radicals, and minimizes amino acid loss. This further ensured no thereby, adverse effects on FI, malnutrition, or growth performance (Hang et al., 2018; Attia et al., 2020).

**Table 3.** Growth performance and breast meat quality of Korat chicken at 9 wk of age

Periods	Levels of TO^1^	Performance parameters				Meat quality					
		BW^2^	BWG^3^	FI^4^	FCR^5^	pH 45 min	pH 24 h	Drip loss (%)	Cooking loss (%)	WBS^6^ (kgf/0.5 cm^2^)	TBARS^7^ (g/kg)
Control^8^		1,134.29	889.27	2,835.78	3.19	5.47	5.68	10.09	26.24	3.25	0.44
3-9 wk of age^9^	1.5%	1,127.05	881.68	2,792.76	3.19	5.51	5.56	9.10	27.53	3.10	0.48
	3.0%	1,112.40	866.66	2,941.27	3.40	5.40	5.61	8.58	26.68	3.15	0.55
	4.5%	1,098.36	852.56	2,851.98	3.34	5.52	5.65	9.36	25.70	3.13	0.62
6-9 wk of age^10^	1.5%	1,098.58	853.34	2,799.49	3.29	5.55	5.62	8.03	24.73	3.08	0.42
	3.0%	1,145.07	898.02	2,849.71	3.18	5.47	5.56	9.01	25.79	3.10	0.54
	4.5%	1,150.00	903.43	2,784.24	3.09	5.59	5.69	7.84	26.56	3.09	0.60
SEM		10.09	9.95	36.26	0.05	0.02	0.02	0.31	0.41	0.10	0.02
*P*-value of contrast											
C vs. other treatment		0.69	0.66	0.99	0.72	0.53	0.18	0.11	0.95	0.64	0.15
Period		0.46	0.47	0.58	0.321	0.19	0.67	0.30	0.31	0.87	0.53
Level of TO		0.864	0.878	0.648	0.89	0.10	0.17	0.96	0.99	0.99	0.07
Period × Level of TO		0.401	0.402	0.899	0.465	0.97	0.52	0.48	0.27	1.00	0.91

^1^1.5% TO, 3.0% TO, and 4.5% TO indicates the group that Korat chicken fed 1.5%, 3.0%, and 4.5% of tuna oil (TO), respectively.

^2^Body weight at 9 wk of age (g).

^3^Body weight gain at 3 to 9 wk of age (g).

^4^Feed intake at 3 to 9 wk (g).

^5^Feed conversion ratio at 3 to 9 wk.

^6^Warner–Bratzler shear force.

^7^Thiobarbituric-acid-reactive substance.

^8^Control indicates the group that Korat chicken fed 4.5% of rice bran oil for 6 wk.

^9^Supplement tuna oil (TO) in each level from 3 to 9 wk.

^10^Supplement tuna oil (TO) in each level from 6 to 9 wk.

Moreover, including vitamin E in the chicken diet not only prevented FA oxidation in feed but also protected against FA oxidation in meat (Vieira et al., 2021), resulting in protection from discoloration and tissue damage, leading to no negative effects on meat quality parameters such as drip loss, cooking loss, nutrient loss, and TBAR value (Abd El-Samee et al., 2019). Therefore, this result shows that dietary TO supplementation for omega-3-enriched meat has no adverse effect on KRC growth performance and meat quality.

### Fatty acids profile of chicken breast meat

The FA profiles of the KRC breast meat were analyzed and are summarized in Table 4. It is known that dietary FA can alter the composition of FA in chicken meat (Skrivan et al., 2018), and in this regard, there are two hypotheses. The first hypothesis is that dietary TO supplementation causes different FA profiles in KRC breast meat when compared with dietary RBO addition, and the second hypothesis is that increasing levels and feeding periods of TO supplementation cause an increase in n-3 PUFA and a decrease in n-6 PUFA accumulation in KRC breast meat.

**Table 4.** Major fatty acid compositions of breast meat supplemented with control and different dietary tuna oil levels and feeding periods^1^

Periods	Levels of TO^2^	Fatty acids										
		C18:2n-6	C20:4n-6	C18:3n-3	C20:5n-3	C22:6n-3	SFA^3^	MUFA^4^	PUFA^5^	n-6^6^	n-3^7^	n-6/n-3 ratio^8^
Control^9^		22.61^a^	11.17^a^	0.32	0.00^e^	1.37^e^	37.59^bc^	26.87^a^	35.46^b^	33.78^a^	1.68^e^	24.91^a^
3 to 9 wk of age^10^	1.5%	18.65^bc^	6.37^b^	0.40	1.64^cd^	9.96^cd^	37.05^bc^	25.81^ab^	37.02^ab^	25.02^bc^	12.00^cd^	2.18^b^
	3.0%	18.65^bc^	4.12^cd^	0.41	2.03^bc^	11.62^bc^	37.76^ab^	25.34^ab^	36.83^ab^	22.78^cd^	14.06^bc^	1.70^b^
	4.5%	15.64^d^	3.53^d^	0.34	3.16^a^	14.68^a^	38.29^ab^	24.24^bc^	37.34^ab^	19.16^e^	18.17^a^	1.09^b^
6 to 9 wk of age^11^	1.5%	20.45^ab^	7.43^b^	0.53	1.50^d^	8.63^d^	35.15^c^	26.31^ab^	38.53^a^	27.87^b^	10.66^d^	2.84^b^
	3.0%	17.53^cd^	6.12^b^	0.89	1.69^cd^	10.37^cd^	36.45^bc^	26.85^a^	36.60^ab^	23.65^cd^	12.95^cd^	1.98^b^
	4.5%	16.23^cd^	5.5b^c^	0.34	2.42^b^	12.64^b^	39.55^a^	23.21^c^	37.15^ab^	21.76^de^	15.40^b^	1.46^b^
SEM		1.729	1.240	0.429	0.335	0.335	1.308	1.556	1.857	1.920	1.530	1.535
*P*-value of contrast												
C vs. other treatment		<0.001	<0.001	0.309	<0.001	<0.001	0.670	0.016	0.015	<0.001	<0.001	<0.001
Period		0.4033	<0.001	0.102	<0.001	0.0002	0.092	0.470	0.498	0.004	0.003	0.327
Level of TO		<0.001	<0.001	0.131	<0.001	<0.001	<0.001	<0.001	0.284	<0.001	<0.001	0.045
Linear		<0.001	<0.001	0.407	<0.001	<0.001	<0.001	<0.001	0.425	<0.001	<0.001	0.027
Quadratic		0.5061	0.123	0.066	0.0033	0.242	0.3167	0.015	0.170	0.682	0.2463	0.904
Period × Level of TO		0.0337	0.4651	0.270	0.0422	0.6521	0.003	0.076	0.327	0.294	0.2561	0.933

^1^Value showed in g/100 g total FA.

^2^1.5% TO, 3.0% TO, and 4.5% TO indicates the group that Korat chicken fed 1.5%, 3.0%, and 4.5% of tuna oil (TO), respectively.

^3^Saturated fatty acid.

^4^Monounsaturated fatty acid.

^5^Polyunsaturated fatty acid.

^6^Omega-6 polyunsaturated fatty acids.

^7^Omega-3 polyunsaturated fatty acids.

^8^Omega-6 to omega-3 polyunsaturated fatty acid ratio.

^9^Control indicates the group that Korat chicken fed 4.5% of rice bran oil for 6 wk.

^10^Supplement tuna oil (TO) in each level from 3 to 9 wk.

^11^Supplement TO in each level from 6 to 9 wk.

^a,b,c,d,e^Value in the same column with different superscripts differ significantly (*P* < 0.05).

Significant differences were observed between the control and treatment groups in various fatty acids, including linoleic acid (**LA**, **C18:2n-6**), arachidonic acid (**AA**, **C20:4n-6**), eicosapentaenoic acid (**EPA**), docosahexaenoic acid (**DHA**), monounsaturated fatty acids (**MUFA**), PUFA, n-3 PUFA, n-6 PUFA, and the ration of n-6 to n-3 PUFA. The breast meat from chickens fed TO exhibited significantly higher percentage of EPA, DHA, and total n-3 PUFA (*P* < 0.01) compared to the control group. Conversely, the control chickens had higher level of LA, AA, n-6 PUFA, and the ratio of n-6 to n-3 PUFA in their breast meat compared to the experimental groups (*P* < 0.01). These findings support our initial hypothesis and align with previous studies indicating that the FA composition of breast meat is primarily influenced by the addition of oil sources and the FA profile of the feed (Skirvan et al., 2018; Attia et al., 2020). In the control group of our study, RBO was used as an energy source, which primarily consisted of oleic acid (C18:1n-9; 41.81%), LA (31.58%), and palmitic acid (C16:0; 21.68%) (Supplementary Table S1). Conversely, TO was used as the source of n-3 PUFA in the experimental treatment, specifically EPA and DHA. This indicates that different dietary fat sources can produce distinct FA profiles in KRC breast meat. Moreover, the KRC that fed the control diet exhibited higher MUFA content compared to the KRC that fed 4.5% TO for both short (6 to 9 wk) and long periods (3 to 9 wk) (*P* = 0.016). Additionally, the control group showed the lowest PUFA content among the groups (*P* = 0.015). Saleh et al. (2009) explained that PUFA can inhibit the activity of delta-9 desaturase, which is responsible for converting precursors into MUFA. This could explain the higher MUFA levels observed in the breast meat of the control group in our study.

The interaction between the level of TO supplementation and feeding period had significant effects on the levels of LA, EPA, and total saturated fatty acid (**SFA**) in KRC breast meat (*P* < 0.05). The group supplemented with 4.5% TO for long period (3 to 9 wk) exhibited the highest EPA content and the lowest LA content compared to the other treatments (*P *< 0.05). In our study, the addition of TO to the treatment groups result in a lower EPA content (11.76%) compared to DHA content (13.89%) (Supplementary Table S1). Previous research has shown that consuming a high amount of dietary DHA can elevate DHA and EPA tissue levels, mediated by peroxisomal oxidation and a group of isomerases and reductase enzymes after 12 wk of feeding (Metherel et al., 2017). Additionally, since n-3 PUFA are more likely to be stored in the phospholipid, EPA and DHA may compete for residency in the membrane’s phospholipids (Pal et al., 2020). In our study, the TO used had a lower EPA content, which could explain the lower EPA level in the experimental diet. It may require a higher proportion of TO and long feeding period to accumulate EPA in KRC breast meat. Furthermore, high amounts of n-3 PUFA have been found to inhibit the desaturase and elongase enzymes of LA (Lopez-Ferrer et al., 2001), which could explain why a high level of TO supplementation for a prolonged periods resulted in the highest EPA content and the lowest LA content. Additionally, we observed that KRC supplemented with 4.5% TO for short periods (6 to 9 wk) exhibited higher SFA levels in breast meat compared to KRC supplemented with 1.5% TO (*P* < 0.05), likely due to the SFA-to-MUFA ratio in feed composition. As indicated in Table 1, the formulation of the 4.5% TO feed had a higher SFA-to-MUFA ratio than the 1.5% TO feed, resulting in a higher proportion of SFA in breast meat of KRC supplemented with 4.5% TO for a short period.

The addition of dietary TO to the chicken diet altered the contents of AA, DHA, MUFA, n-6 PUFA, n-3 PUFA, and the n-6 to n-3 ratio in breast meat (*P* < 0.05). The increasing level of TO supplementation linearly decreased AA (*P* < 0.001), MUFA (*P *< 0.001 for linear; *P* = 0.015 for quadratic), n-6 PUFA (*P* < 0.001), and n-6 to n-3 ratio (*P* < 0.001), while it linearly increased DHA (*P* < 0.001), and n-3 PUFA (*P* < 0.001). The results of this investigation are consistent with those of previous studies, which showed that increasing the levels of n-3 PUFA in chicken diets enhanced n-3 PUFA and reduced the content of n-6 PUFA in chicken meat (Farhoomand and ­Checaniazer 2009; Yang et al., 2010). The associations between n-3 PUFA and n-6 PUFA contents in the meat and the level of TO supplementation can be explained by the competition of desaturase and elongase enzymes between n-6 and n-3 PUFA (Cortinas et al., 2004). High levels of n-3 PUFA may have reduced the desaturase and elongase enzymes of the n-6 PUFA family (Lopez-Ferrer et al., 2001). The consumption of food with a high ratio of n-6 to n-3 PUFA can increase the risk of cardiovascular disease and cancer. In the secondary protection of cardiovascular disease, Simopoulos (2004) reported that the 1 to 4 ratio of n-6 to n-3 PUFA was associated with a 70% reduction in total mortality. In this study, the group supplemented with TO showed an n-6 to n-3 ratio less than 4 in KRC breast meat. Furthermore, a significant TO addition to the chicken diet resulting in increased DHA in chicken meat could be due to the high amount of DHA in the KRC diet, and in the final process of FA biosynthesis, DHA can be produced by chain elongation, delta-6- desaturase activity, and peroxisomal β-oxidation from EPA (Poureslami et al., 2010).

Interestingly, the period of TO supplementation significantly affected the proportion of AA, DHA, n-3 PUFA, and n-6 PUFA in KRC breast meat (*P* < 0.05). KRC ­supplemented with the TO diet for long period (3 to 9 wk) exhibited higher proportions of DHA and n-3 PUFA but lower proportion of AA and n-6 PUFA compared to KRC supplemented with TO diet for a shorter period (6 to 9 wk). These findings align with the results of Konieczka et al. (2017), who observed increased DHA content in breast meat with a longer feeding period of flaxseed and FO. Our study found that KRC fed 4.5% TO for a short period, resulted in 260.75 mg of EPA + DHA/100 g fresh meat, as calculated using the formula reported by Hang et al. (2018). This level corresponds to achieving a “high in n-3 PUFA” classification according to the recommendations for diets described by the Commission Regulations (EU) 1924/2006 (Verhagen et al., 2010). These results are consistent with the findings of Zuidhof et al. (2009), who demonstrated that supplementing the diet with ground full-fat flaxseed for 24.1 d was a cost-effective approach to achieve n-3 PUFA-enriched-chicken meat. Furthermore, Kanakri et al. (2017) added flaxseed to broiler diets and found that feeding for 2 to 4 wk before slaughter was sufficient for chickens to accumulate the same amount of n-3 PUFA as feeding for 6 wk. This suggests that a diet supplemented with 4.5% TO for a short period is adequate to enhance the n-3 PUFA content in KRC breast meat.

### Correlation loadings plot of PCA of fatty acids from different supplementation of TO levels and feeding periods

In this study, we investigated the FA profile of KRC breast meat, focusing on key FA, particularly the group of n-3 PUFA, that are abundant in TO oil and used as a supplement in the experimental diet. These n-3 PUFA have been recognized for their beneficial effects on various biological, physiological, developmental, reproductive, and health aspect in poultry and humans (Alagawany et al., 2019). PCA was employed to explore the relationship between the levels and periods of TO supplementation and the key FA in KRC breast meat.

The results of the score plot and correlation loading of n-3 PUFA and n-6 PUFA groups in KRC breast meat are shown in Figure 1A and 1B. The FA profiles of KRC breast meat from different treatment groups exhibited distinct separations, accounting for 89% of the total variation in the data sets. Notably, there was a clear distinction between the control group, T4 and T2, T3, and T6 (Figure 1A). However, an overlap was observed between T1 and T5, positioned in the middle of the PC-1 axis in the score plot, indicating no significant association with the traits of interest.

All variables within the outer circle region (EPA, DHA, n-3 PUFA; LA, AA, n-6 PUFA, and n-6 to n-3 PUFA) displayed significant correlations with the seven experimental groups, accounting for over 50% of the variance (Figure 1B). T2, T6, and particularly T3 exhibited positive correlations with EPA, DHA, and n-3 PUFA but negative correlations with AA, LA, n-6 PUFA, and the n-6 to n-3 PUFA ratio. These findings suggest that KRC fed a TO diet with 3.0% supplementation for a long period (3 to 9 wk) and a TO diet with 4.5% supplementation for a short period (6 to 9 wk) or a long period (3 to 9 wk) exhibited a higher level of EPA, DHA, and n-3 PUFA content while demonstrating lower levels of n-6 PUFA, and the n-6 to n-3 PUFA ratio in their breast meat. Notably, the group receiving 4.5% TO supplementation for a long period (3 to 9 wk) results in the highest content of EPA, DHA, and n-3 PUFA content, along with the lowest content of n-6 PUFA and the n-6 to n-3 PUFA ratio in their breast meat. On the other hand, group T4 and especially the control group exhibited positive correlation with AA, LA, n-6 PUFA, and the n-6 to n-3 PUFA ratio but negative correlation with EPA, DHA, and n-3 PUFA. These results indicated that KRC fed the ­control diet or a TO 1.5% supplementation for a long period (3 to 9 wk) showed higher level of the n-6 PUFA group and the n-6 to n-3 ratio while accumulating lower levels of the n-3 PUFA group in their breast meat. In particular, the control group exhibited the highest level of AA, LA, n-6 PUFA, and the n-6 to n-3 PUFA ratio, while the n-3 PUFA group exhibited the lowest level in breast meat. These findings are consistent with the FA composition of KRC breast meat presented in Table 4, confirming the results obtained through PCA.

### L-FABP, PPARA, and LPL gene expression

We hypothesized that chickens supplemented with TO for a long period, with high levels of key FA such as EPA and DHA, would enhance the expression of genes involved in lipid transport, oxidation, and conversion of tetracosahexaenoic acid to DHA, as well as activate the *LPL* gene expression, involved in lipid storage in chicken meat. However, most of the results related to genes involved in lipid metabolism did not support our hypothesis, except for the expression of the *L-FABP* gene in the liver.

The interaction between the level and feeding periods of TO supplementation significantly affected *PPARA* gene expression in the liver and *LPL* gene expression in the breast (*P* < 0.001). The TO supplementation level also affected *L-FABP* gene expression in the liver (*P* < 0.001) (Table 5).

*L-FABP* plays a role in lipids’ metabolism and intracellular transportation, exhibiting a high affinity for long-chain fatty acids (**LCFA**). The increasing level of TO supplementation showed a linear increased in *L-FABP* gene expression in the liver (*P* < 0.001 for linear; *P* = 0.032 for quadratic). This finding is consistent with the reports of Atshaves et al. (2010) and can be attributed to the consumption of TO, a rich source of PUFA, including EPA and DHA, know to induce the expression of *L-FABP*. Furthermore, Newberry et al. (2009) demonstrated that *L-FABP* is involved in FA metabolism and the uptake of LCFA, transporting them to complex lipids for membrane synthesis and storage. In contrast, the highest levels of *PPARA* gene expression were observed in the livers of KRC that received 3.0% TO for a short period (6 to 9 wk). Previous studies have reported that PUFA, EPA, and DHA activate *PPARA* expression (Berge et al., 1999; Clarke, 2000; Royan et al., 2011) in various species, including broiler chicken, turkeys (Ding et al., 2003), mice (Issemann and Green, 1990), and humans (Schmidt et al., 1992). However, in our study, increasing levels and periods of TO supplementation did not lead to an increase in *PPARA* gene expression. This discrepancy may be attributed to the different types of FA present in the treatments, as different FA and n-3 to n-6 PUFA ratios can activate different isoforms of PPARs. PPARs are known to be encoded by PPARs genes and activated by different FA with varying affinity to the receptors (Dubey and Cheema, 2006). The three isoforms of PPARs, namely PPARα, β/δ, and γ, control different target genes. Thus, the presence of different FA and varied n-3 to n-6 PUFA ratios in the different treatments may have led to the activation of different PPARs isoforms, resulting in inconsistent results with our initial hypothesis. In addition, the highest *LPL* gene expression detected in chickens received 3.0% TO for a long period (3 to 9 wk). However, Yan and Chao (2011) reported that *LPL* expression was reduced by DHA intake, and many factors are thought to influence *LPL* expression, including age, breed, and the FA content of the skeletal muscle in chickens.

### The correlation loadings plot between control, and treatments (the combination between levels and periods of TO supplementation), and gene expression in the breast meat of male KRC

The score plot and correlation loading, illustrating the relationship between the levels and periods of TO supplementation and gene expression in KRC breast meat, are presented in Figure 2A and 2B.

In the score plot, the expression patterns of *L-FABP*, *PPARA* in the liver, and *LPL* in KRC breast meat from the control and different treatment groups are clearly separated, accounting for 84% of the total variation. The control and treatment groups are distinct from each other. The correlation loading plot in Figure 2A displays the expression level of *L-FABP*, *PPARA,* and *LPL* genes within the outer circle areas, explaining over 50% of the variance among the seven groups and demonstrating significant correlations among these traits. The control group is positioned at the center of the PC-1 axis in the score plot, indicating a lack of correlation between the control and all the gene expressions include in the analysis. However, T1 and T5 exhibited a positive correlation with *PPARA* gene expression. Moreover, groups T3 and T6 display a positive correlation with *L-FABP* gene expression, while groups T2 and T4 show a positively correlated with *LPL* gene expression. These findings from the PCA analysis align with some of the results as shown in Table 5.

**Table 5.** Expression of the *L-FABP*, *PPARA*, and *LPL* at 9 wk of age^1^

Periods	Levels of TO^2^	Expression level		
		*PPARA* ^3^	*LPL* ^4^	*L-FABP* ^5^
Control^6^		1.29^bc^	0.94^bc^	0.93^c^
3 to 9 wk of age^7^	1.5%	1.67^ab^	0.84^c^	0.51^c^
	3.0%	0.97^c^	1.75^a^	1.02^bc^
	4.5%	1.16^bc^	0.61^c^	2.43^a^
6 to 9 wk of age^8^	1.5%	0.87^c^	1.29^b^	0.57^c^
	3.0%	1.88^a^	0.83^c^	0.86^c^
	4.5%	1.15^bc^	0.87^bc^	1.91^ab^
SEM		0.539	0.193	0.417
*P*-value of contrast				
C vs. other treatment		0.949	0.383	0.219
Period		0.755	0.401	0.241
Level of TO		0.116	<0.001	<0.001
Linear		0.365	0.003	<0.001
Quadratic		0.061	0.001	0.032
Period × Level of TO		<0.001	<0.001	0.390

^1^Four individuals were used (*n* = 4).

^2^1.5% TO, 3.0% TO, and 4.5% TO indicates the group that Korat chicken fed 1.5%, 3.0%, and 4.5% of tuna oil, respectively.

^3^Peroxisome proliferator-activated receptor alpha gene expression.

^4^Lipoprotein lipase.

^5^Liver fatty acid-binding protein.

^6^Control indicates the group that Korat chicken fed 4.5% of rice bran oil for 6 wk.

^7^Supplement tuna oil (TO) in each level from 3 to 9 wk.

^8^Supplement TO in each level from 6 to 9 wk.

^a,b,c^Value in the same column with different superscripts differ significantly (*P *< 0.05).

### The correlation between gene expression and key FA content in breast meat

From the previous section, our results prove that different levels of supplements and feeding periods of TO affect FA profiles in KRC breast meat and genes related to fat metabolism (Tables 4 and 5). These results raise the question of the relationship between FA composition in KRC breast meat and gene expression and how it changes after KRC receives feed containing TO.

To address this question, we generated a correlation matrix to explore the relationship.

**Figure 3.**
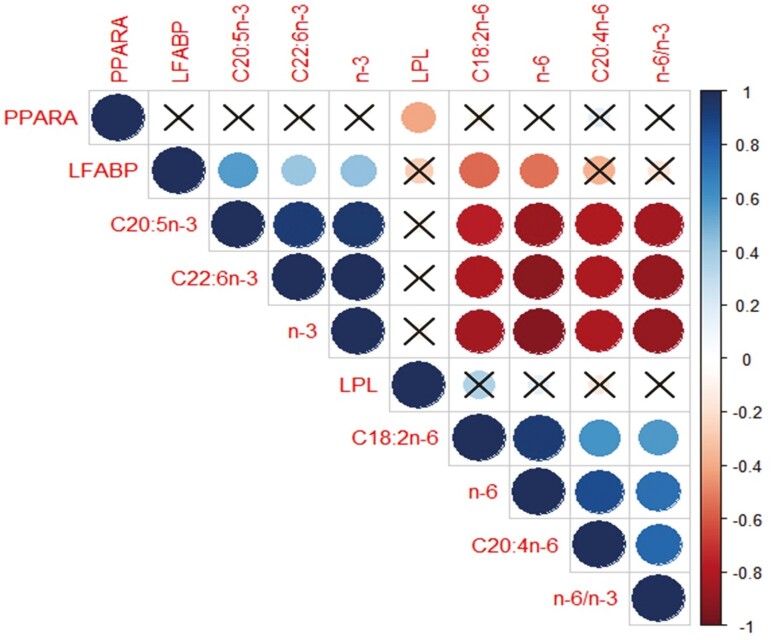
The correlation matrix between gene expression and key FA content in KRC meat. Blue and large circles mean a strongly positive correlation, whereas red and large circle represent a strongly negative correlation. −1 indicates a perfectly negative linear correlation between two variables. 0 indicates no linear correlation between two variables. 1 indicates a perfectly positive linear correlation between two variables. ** a circle without crisscross symbol (×) means correlations significant at the *P *< 0.05 level. ** a circle with crisscross symbol (×) means no correlation. Abbreviation: C20:5n-3, eicosapentaenoic acid (EPA); C22:6n-3, docosahexaenoic acid (DHA); C18:2n-6, linoleic acid (LA); C20:4n-6, arachidonic acid (AA); n-3, omega-3 polyunsaturated fatty acids (n-3 PUFA); n-6, omega-6 polyunsaturated fatty acids; n-6/n-3, n-6 to n-3 PUFA ratio; LPL, lipoprotein lipase gene; PPARA, peroxisome proliferator-activated receptor alpha; L-FABP, liver fatty acid-binding proteins.

The correlation matrix (Fig. 3) revealed interesting relationships between the concentrations of EPA, DHA, and n-3 PUFA in KRC breast meat and gene expression. *L-FABP* gene expression showed a positive correlation with the levels of EPA, DHA, and n-3 PUFA while exhibiting a strongly negative correlation with the levels of n-6 PUFA, including AA, LA, and an n-6 to n-3 ratio. Conversely, AA, LA, n-6 PUFA, and an n-6 to n-3 ratio in breast meat exhibited a strong negative correlation with the concentrations of EPA, DHA, and n-3 PUFA content. Also, LA and n-6 PUFA negatively correlated with *L-FABP* gene expression. In the liver, *L-FABP* gene expression demonstrated a robust positive correlation with EPA, DHA, and n-3 PUFA while displaying a negative correlation with LA and n-6 PUFA. These findings suggest a relationship between *L-FABP* gene expression and the FA composition in KRC breast meat. The upregulation of *L-FABP* gene expression in KRC livers likely contributes to increased levels of FA, particularly EPA and DHA. These FA are predominantly transported to mitochondria or peroxisomes, enhancing FA acid oxidation and reducing fat deposition (Wang et al., 2006). Additionally, DHA can be retro-converted to EPA via B-oxidation in mitochondria or peroxisomes (Park et al., 2016). Consequently, the supplementation of DHA in the chicken diet resulted in reduced abdominal fat, as observed in the group of KRC fed 4.5% TO (data not shown), and increased accumulation of EPA and DHA. These effects are likely attributed to the direct modification of FA composition through dietary PUFA and the retroversion process occurring in mitochondria and peroxisomes.

Interestingly, a strong negative correlation was observed between *PPARA* gene expression in the liver and *LPL* gene expression in breast meat, contradicting previous studies that have reported a direct control of *LPL* gene expression by PPARA through a functional peroxisome proliferator hormone response element in its promoter region (Schoonjans et al., 1996). The relationship between *PPARA* and *LPL* gene expression has been studied in various animal models, such as lactating mice, where downregulation of PPARA has been shown to affect FA and triacylglycerol synthesis, resulting in altered *LPL* gene expression and FA uptake in different tissues (Grigor and warren, 1980; Gutgesell et al., 2009). The regulation of *LPL* gene expression is complex and involves multiple factors, nutrient status, hormonal levels, and stress levels (Wang and Eckel, 2009). Furthermore, various genes and factors influence the regulation of FA metabolism and accumulation in chicken meat. Therefore, future research should focus on investigating all genes associated with fat metabolism in KRC to understand the underlying mechanisms and utilize them as markers for enhancing the value and quality of KRC meat. Unfortunately, this study found no significant relationship between *PPARA* and *LPL* gene expression and FA content in KRC breast meat.

In conclusion, this study investigated the effects of dietary TO levels and feeding periods of TO supplementation on various aspects of slow-growing KRC, including growth performance, meat quality, omega-3 FA profile, and gene expression related to FA metabolism. The results revealed that the interaction between TO levels and feeding periods did not significantly impact the growth performance and meat quality of KRC. However, when KRC was fed 4.5% TO for a long period, their breast meat exhibited the highest level of EPA and the lowest levels of LA. Moreover, increasing the level of TO supplementation resulted in a linear decrease in AA, MUFA, n-6 PUFA, and the n-6 to n-3 PUFA ratio while linearly increasing DHA, n-3 PUFA, and *L-FABP* gene expression. Notably, a positive correlation was observed between the concentrations of EPA, DHA, and n-3 PUFA and *L-FABP* gene expression. However, no significant relationship was found between FA and *PPARA* or *LPL* gene expression. Furthermore, KRC that fed a TO-supplemented diet for a long period (3 to 9 wk) had higher proportions of DHA and n-3 PUFA but lower proportions of AA and n-6 PUFA compare to those fed the diet for a short period. It is worth mentioning that the KRC breast meat from chickens fed the 4.5% TO for both short and long periods contained at least 80 mg of EPA + DHA per 100 g of meat, meeting the criteria for being “high in n-3 PUFA” as defined in the dietary recommendations. Therefore, the findings suggest that supplementing the diet with 4.5% TO for a short period produces n-3 PUFA-enriched meat in slow-growing chickens.

This study indicates that supplementing slow-growing chickens with omega-3-rich TO can improve the FA profile of their meat, boosting its nutritional value by increasing beneficial FA levels. Interestingly, even a short period of TO supplementation can achieve desirable omega-3 enrichment in slow-growing chicken meat without compromising growth performance and meat quality. This finding has practical implications in the poultry industry, providing insights into the optimal level and period of TO supplementation for developing an effective feeding scheme to enhance n-3 PUFA in chicken meat.

## Supplementary Material

skad267_suppl_Supplementary_Table_S1

## Abbreviations

AA: arachidonic acid
ADG: average daily gain
BW: body weight
BWG: Body weight gain
CP: crude protein
DHA: docosahexaenoic acid
EPA: eicosapentaenoic acid
FA: fatty acid
FADS1: fatty acid desaturase 1
FADS2: fatty acid desaturase 2
FCR: feed conversion ratio
FI: feed intake
FO: fish oil
GAPDH: Glyceraldehyde-3-phosphate dehydrogenase
KRC: Korat chicken
L-FABP: liver fatty acid-binding proteins
LA: linoleic acid
LCFA: long-chain fatty acids
LHK: Leung Hang Khao
LPL: lipoprotein lipase
MDA: malondialdehyde
n-3 PUFA: omega-3 polyunsaturated fatty acids
n-6 PUFA: omega-6 polyunsaturated fatty acids
MUFA: monounsaturated fatty acids
PCA: principal component analysis
PCR: polymerase chain reaction
PPARs: peroxisome proliferator-activated receptor
PPARA: peroxisome proliferator-activated receptor alpha
PUFA: polyunsaturated fatty acid
RBO: rice bran oil
SDG: sustainable development goal
SFA: saturated fatty acid
SUT: Suranaree University of Technology
TAG: triacylglycerol
TBARS: thiobarbituric-acid-reactive substance
TO: tuna oil
